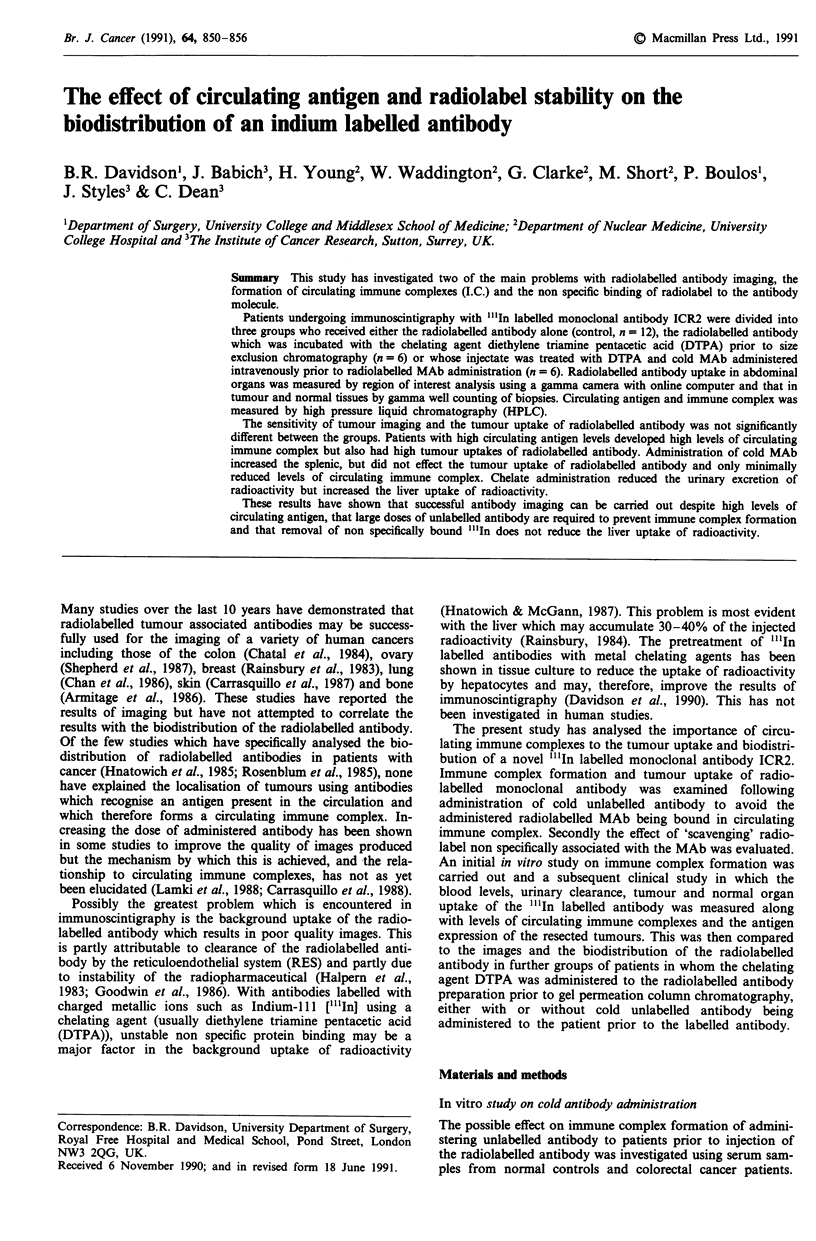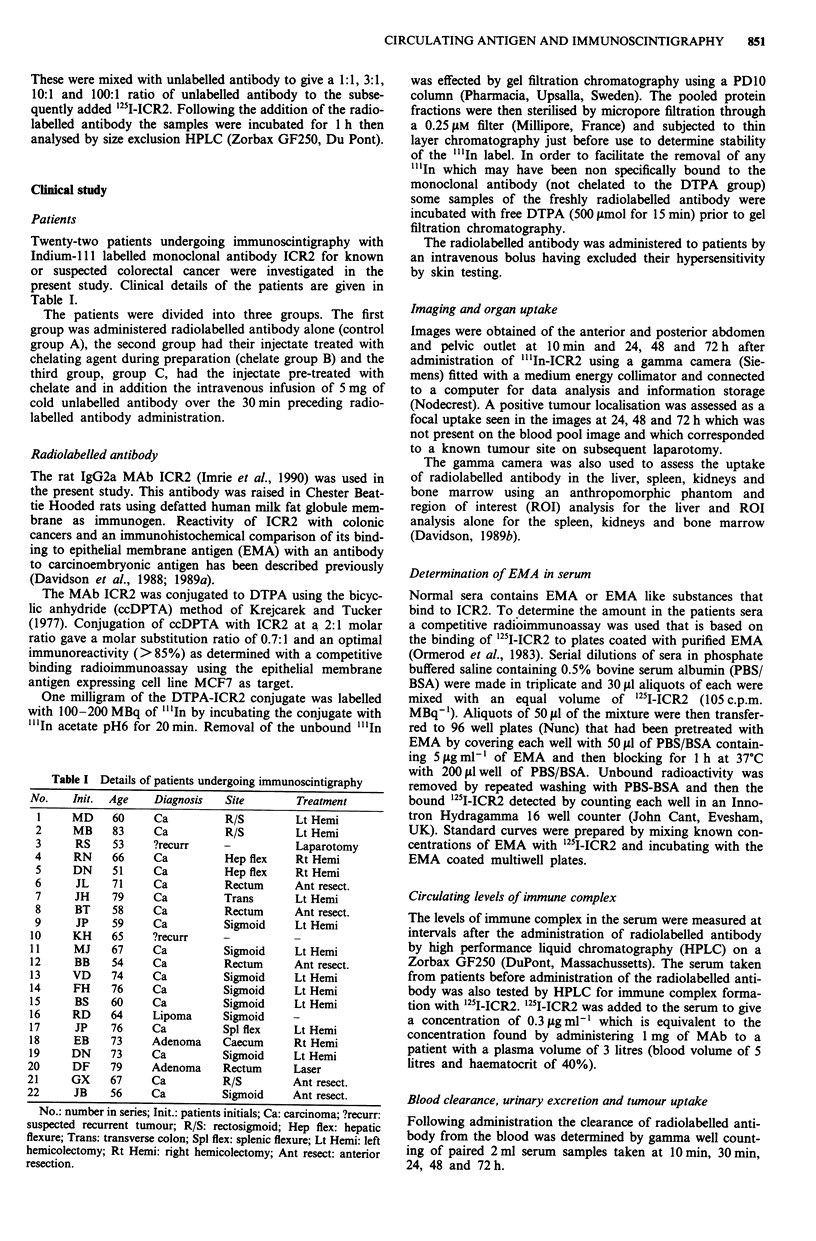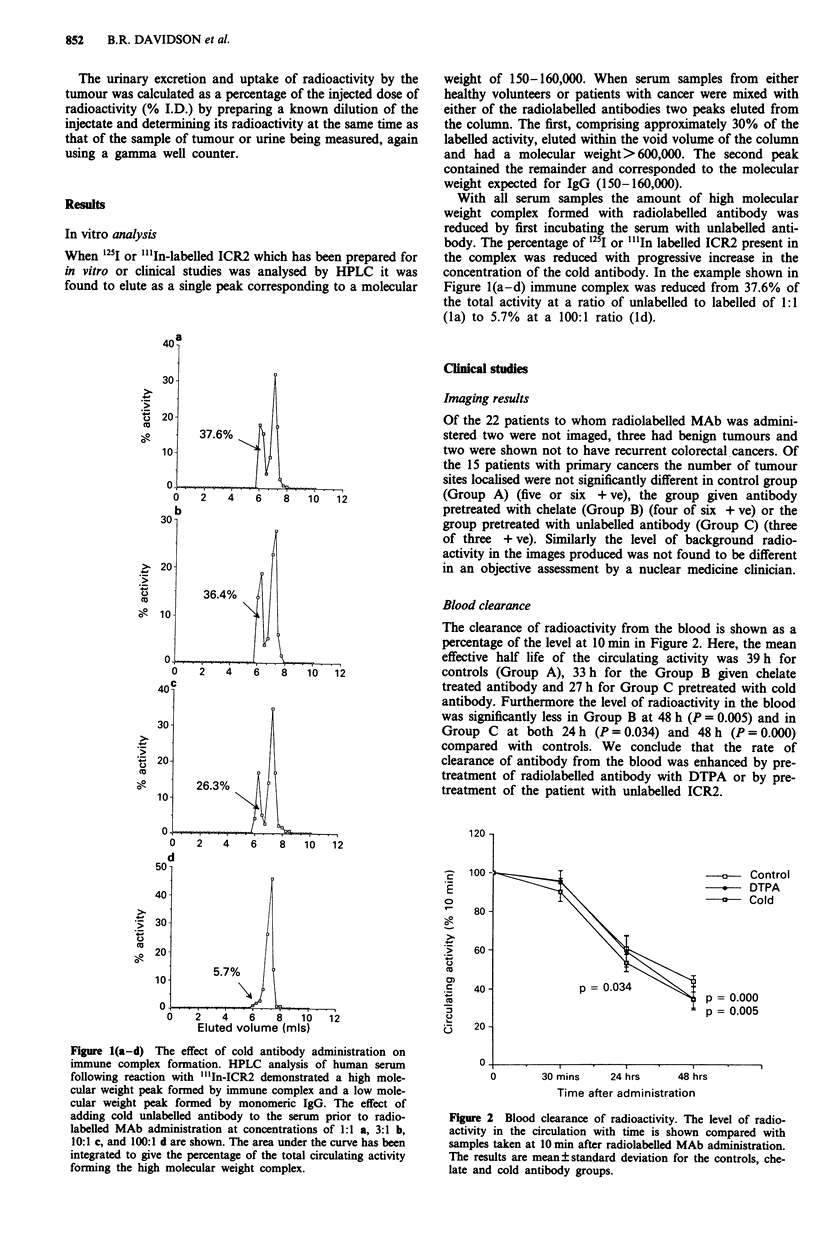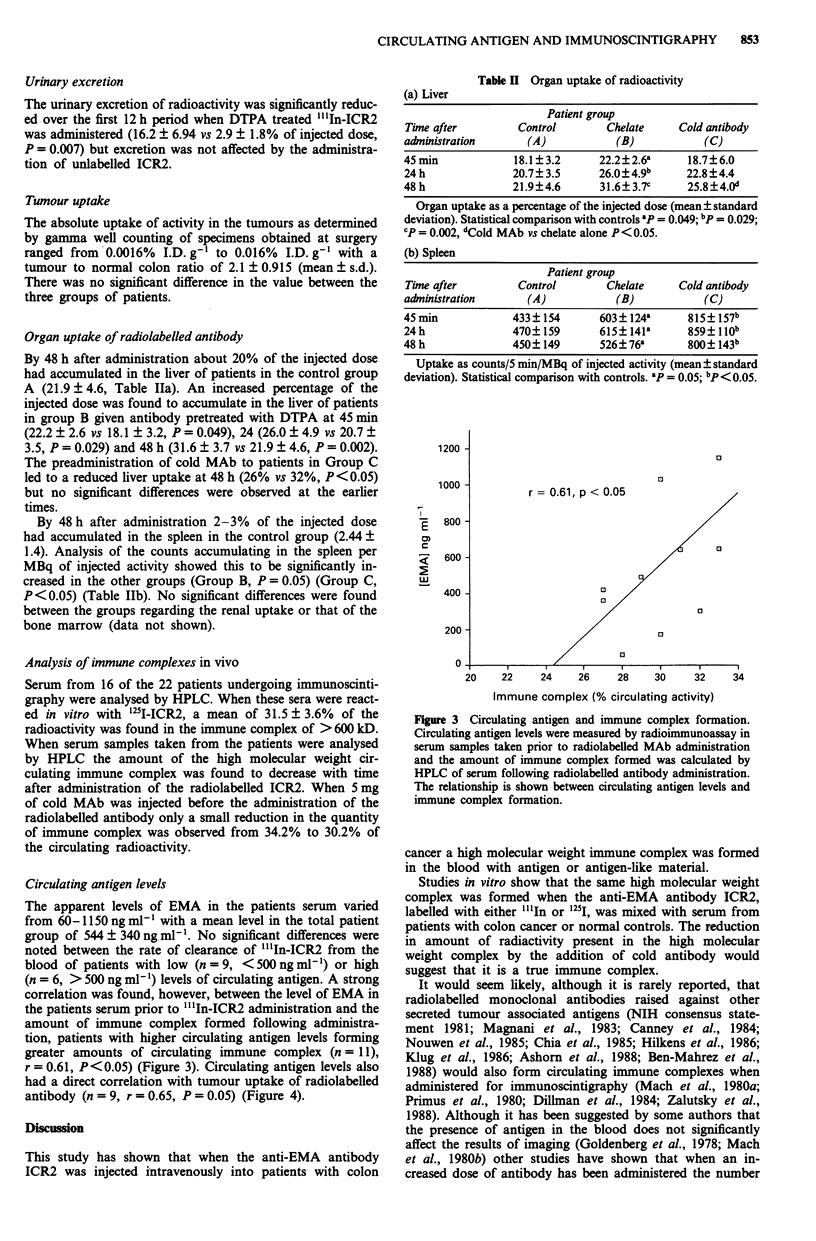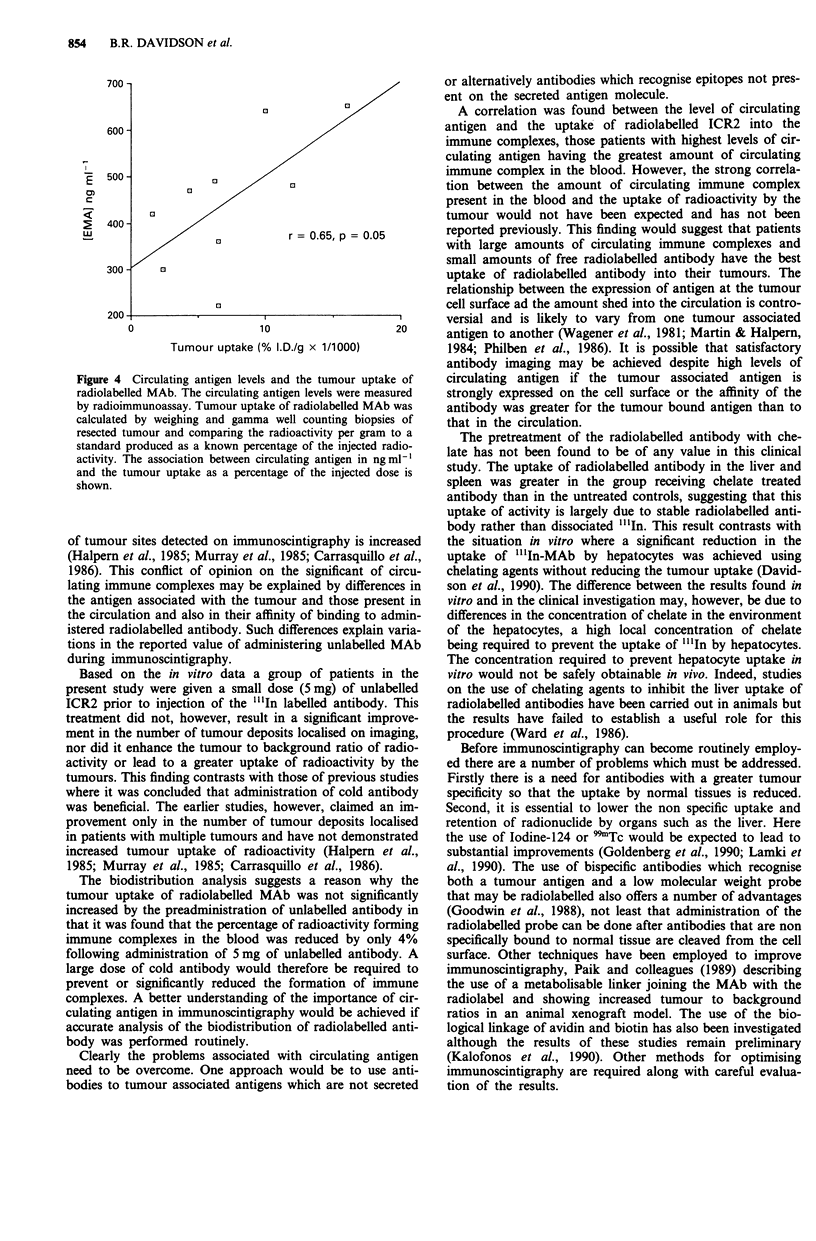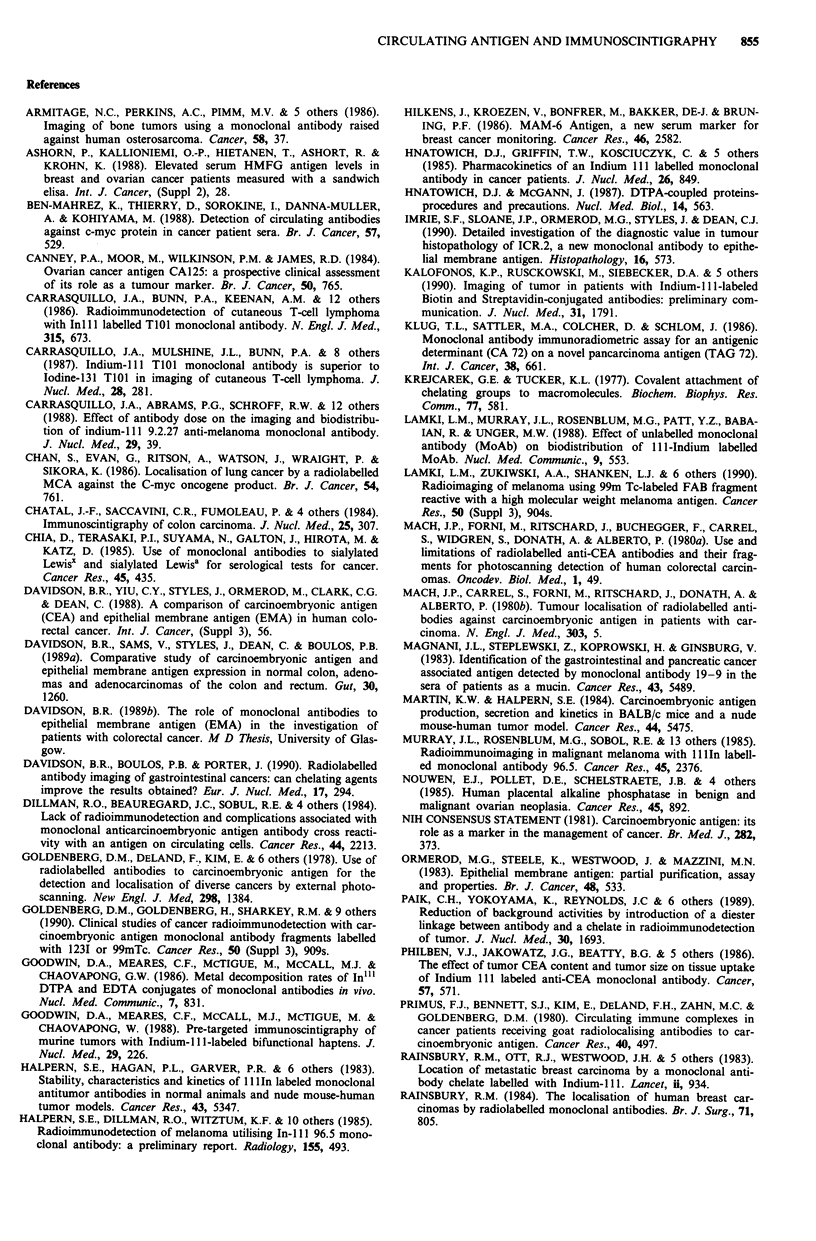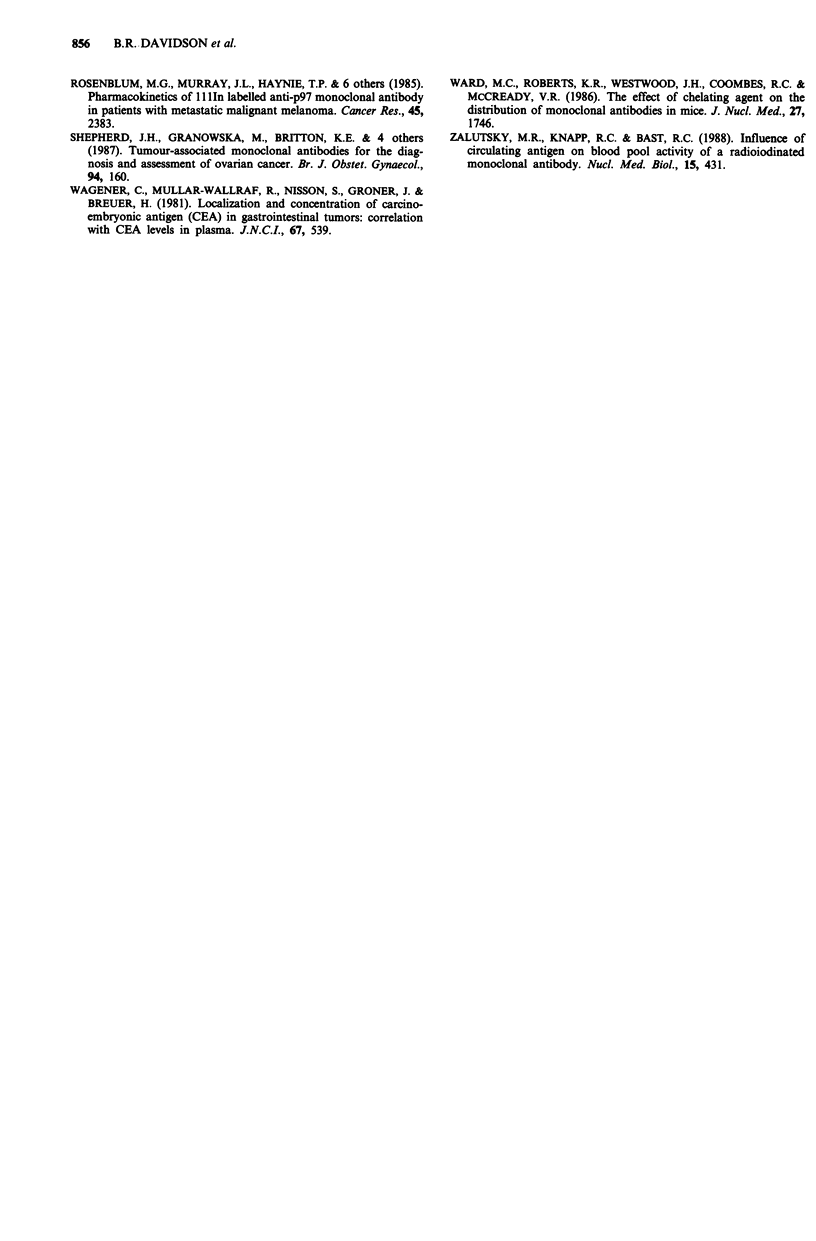# The effect of circulating antigen and radiolabel stability on the biodistribution of an indium labelled antibody.

**DOI:** 10.1038/bjc.1991.412

**Published:** 1991-11

**Authors:** B. R. Davidson, J. Babich, H. Young, W. Waddington, G. Clarke, M. Short, P. Boulos, J. Styles, C. Dean

**Affiliations:** Department of Surgery, University College and Middlesex School of Medicine, Sutton, Surrey, UK.

## Abstract

This study has investigated two of the main problems with radiolabelled antibody imaging, the formation of circulating immune complexes (I.C.) and the non specific binding of radiolabel to the antibody molecule. Patients undergoing immunoscintigraphy with 111In labelled monoclonal antibody ICR2 were divided into three groups who received either the radiolabelled antibody alone (control, n = 12), the radiolabelled antibody which was incubated with the chelating agent diethylene triamine pentacetic acid (DTPA) prior to size exclusion chromatography (n = 6) or whose injectate was treated with DTPA and cold MAb administered intravenously prior to radiolabelled MAb administration (n = 6). Radiolabelled antibody uptake in abdominal organs was measured by region of interest analysis using a gamma camera with online computer and that in tumour and normal tissues by gamma well counting of biopsies. Circulating antigen and immune complex was measured by high pressure liquid chromatography (HPLC). The sensitivity of tumour imaging and the tumour uptake of radiolabelled antibody was not significantly different between the groups. Patients with high circulating antigen levels developed high levels of circulating immune complex but also had high tumour uptakes of radiolabelled antibody. Administration of cold MAb increased the splenic, but did not effect the tumour uptake of radiolabelled antibody and only minimally reduced levels of circulating immune complex. Chelate administration reduced the urinary excretion of radioactivity but increased the liver uptake of radioactivity. These results have shown that successful antibody imaging can be carried out despite high levels of circulating antigen, that large doses of unlabelled antibody are required to prevent immune complex formation and that removal of non specifically bound 111In does not reduce the liver uptake of radioactivity.


Br. J. Cancer (1991), 64, 850-856 © Macmillan Press Ltd., 1991

### The effect of circulating antigen and radiolabel stability on the biodistribution of an indium labelled antibody

B.R. Davidson[1], J. Babich[3], H. Young[2], W. Waddington[2], G. Clarke[2], M. Short[2], P. Boulos[1], J. Styles[3] & C. Dean[3]

[1]Department of Surgery, University College and Middlesex School of Medicine; [2]Department of Nuclear Medicine, University College Hospital and [3]The Institute of Cancer Research, Sutton, Surrey, UK.

**Summary** This study has investigated two of the main problems with radiolabelled antibody imaging, the formation of circulating immune complexes (I.C.) and the non specific binding of radiolabel to the antibody molecule.

Patients undergoing immunoscintigraphy with $^{111}$In labelled monoclonal antibody ICR2 were divided into three groups who received either the radiolabelled antibody alone (control, $n = 12$), the radiolabelled antibody which was incubated with the chelating agent diethylene triamine pentacetic acid (DTPA) prior to size exclusion chromatography ($n = 6$) or whose injectate was treated with DTPA and cold MAb administered intravenously prior to radiolabelled MAb administration ($n = 6$). Radiolabelled antibody uptake in abdominal organs was measured by region of interest analysis using a gamma camera with online computer and that in tumour and normal tissues by gamma well counting of biopsies. Circulating antigen and immune complex was measured by high pressure liquid chromatography (HPLC).

The sensitivity of tumour imaging and the tumour uptake of radiolabelled antibody was not significantly different between the groups. Patients with high circulating antigen levels developed high levels of circulating immune complex but also had high tumour uptakes of radiolabelled antibody. Administration of cold MAb increased the splenic, but did not effect the tumour uptake of radiolabelled antibody and only minimally reduced levels of circulating immune complex. Chelate administration reduced the urinary excretion of radioactivity but increased the liver uptake of radioactivity.

These results have shown that successful antibody imaging can be carried out despite high levels of circulating antigen, that large doses of unlabelled antibody are required to prevent immune complex formation and that removal of non specifically bound $^{111}$In does not reduce the liver uptake of radioactivity.

Many studies over the last 10 years have demonstrated that radiolabelled tumour associated antibodies may be successfully used for the imaging of a variety of human cancers including those of the colon (Chatal *et al.*, 1984), ovary (Shepherd *et al.*, 1987), breast (Rainsbury *et al.*, 1983), lung (Chan *et al.*, 1986), skin (Carrasquillo *et al.*, 1987) and bone (Armitage *et al.*, 1986). These studies have reported the results of imaging but have not attempted to correlate the results with the biodistribution of the radiolabelled antibody. Of the few studies which have specifically analysed the biodistribution of radiolabelled antibodies in patients with cancer (Hnatowich *et al.*, 1985; Rosenblum *et al.*, 1985), none have explained the localisation of tumours using antibodies which recognise an antigen present in the circulation and which therefore forms a circulating immune complex. Increasing the dose of administered antibody has been shown in some studies to improve the quality of images produced but the mechanism by which this is achieved, and the relationship to circulating immune complexes, has not as yet been elucidated (Lamki *et al.*, 1988; Carrasquillo *et al.*, 1988).

Possibly the greatest problem which is encountered in immunoscintigraphy is the background uptake of the radiolabelled antibody which results in poor quality images. This is partly attributable to clearance of the radiolabelled antibody by the reticuloendothelial system (RES) and partly due to instability of the radiopharmaceutical (Halpern *et al.*, 1983; Goodwin *et al.*, 1986). With antibodies labelled with charged metallic ions such as Indium-111 [$^{111}$In] using a chelating agent (usually diethylene triamine pentacetic acid (DTPA)), unstable non specific protein binding may be a major factor in the background uptake of radioactivity (Hnatowich & McGann, 1987). This problem is most evident with the liver which may accumulate 30–40% of the injected radioactivity (Rainsbury, 1984). The pretreatment of $^{111}$In labelled antibodies with metal chelating agents has been shown in tissue culture to reduce the uptake of radioactivity by hepatocytes and may, therefore, improve the results of immunoscintigraphy (Davidson *et al.*, 1990). This has not been investigated in human studies.

The present study has analysed the importance of circulating immune complexes to the tumour uptake and biodistribution of a novel $^{111}$In labelled monoclonal antibody ICR2. Immune complex formation and tumour uptake of radiolabelled monoclonal antibody was examined following administration of cold unlabelled antibody to avoid the administered radiolabelled MAb being bound in circulating immune complex. Secondly the effect of 'scavenging' radiolabel non specifically associated with the MAb was evaluated. An initial *in vitro* study on immune complex formation was carried out and a subsequent clinical study in which the blood levels, urinary clearance, tumour and normal organ uptake of the $^{111}$In labelled antibody was measured along with levels of circulating immune complexes and the antigen expression of the resected tumours. This was then compared to the images and the biodistribution of the radiolabelled antibody in further groups of patients in whom the chelating agent DTPA was administered to the radiolabelled antibody preparation prior to gel permeation column chromatography, either with or without cold unlabelled antibody being administered to the patient prior to the labelled antibody.

### Materials and methods

### In vitro *study on cold antibody administration*

The possible effect on immune complex formation of administering unlabelled antibody to patients prior to injection of the radiolabelled antibody was investigated using serum samples from normal controls and colorectal cancer patients.

Correspondence: B.R. Davidson, University Department of Surgery, Royal Free Hospital and Medical School, Pond Street, London NW3 2QG, UK.
Received 6 November 1990; and in revised form 18 June 1991.

These were mixed with unlabelled antibody to give a 1:1, 3:1, 10:1 and 100:1 ratio of unlabelled antibody to the subsequently added $^{125}$I-ICR2. Following the addition of the radiolabelled antibody the samples were incubated for 1 h then analysed by size exclusion HPLC (Zorbax GF250, Du Pont).

### Clinical study

### *Patients*

Twenty-two patients undergoing immunoscintigraphy with Indium-111 labelled monoclonal antibody ICR2 for known or suspected colorectal cancer were investigated in the present study. Clinical details of the patients are given in Table I.

The patients were divided into three groups. The first group was administered radiolabelled antibody alone (control group A), the second group had their injectate treated with chelating agent during preparation (chelate group B) and the third group, group C, had the injectate pre-treated with chelate and in addition the intravenous infusion of 5 mg of cold unlabelled antibody over the 30 min preceding radiolabelled antibody administration.

### *Radiolabelled antibody*

The rat IgG2a MAb ICR2 (Imrie *et al.*, 1990) was used in the present study. This antibody was raised in Chester Beattie Hooded rats using defatted human milk fat globule membrane as immunogen. Reactivity of ICR2 with colonic cancers and an immunohistochemical comparison of its binding to epithelial membrane antigen (EMA) with an antibody to carcinoembryonic antigen has been described previously (Davidson *et al.*, 1988; 1989*a*).

The MAb ICR2 was conjugated to DTPA using the bicyclic anhydride (ccDPTA) method of Krejcarek and Tucker (1977). Conjugation of ccDPTA with ICR2 at a 2:1 molar ratio gave a molar substitution ratio of 0.7:1 and an optimal immunoreactivity (>85%) as determined with a competitive binding radioimmunoassay using the epithelial membrane antigen expressing cell line MCF7 as target.

One milligram of the DTPA-ICR2 conjugate was labelled with 100–200 MBq of $^{111}$In by incubating the conjugate with $^{111}$In acetate pH6 for 20 min. Removal of the unbound $^{111}$In was effected by gel filtration chromatography using a PD10 column (Pharmacia, Upsalla, Sweden). The pooled protein fractions were then sterilised by micropore filtration through a 0.25 μM filter (Millipore, France) and subjected to thin layer chromatography just before use to determine stability of the $^{111}$In label. In order to facilitate the removal of any $^{111}$In which may have been non specifically bound to the monoclonal antibody (not chelated to the DTPA group) some samples of the freshly radiolabelled antibody were incubated with free DTPA (500 μmol for 15 min) prior to gel filtration chromatography.

The radiolabelled antibody was administered to patients by an intravenous bolus having excluded their hypersensitivity by skin testing.

**Table I** Details of patients undergoing immunoscintigraphy

| *No.* | *Init.* | *Age* | *Diagnosis* | *Site* | *Treatment* |
|---|---|---|---|---|---|
| 1 | MD | 60 | Ca | R/S | Lt Hemi |
| 2 | MB | 83 | Ca | R/S | Lt Hemi |
| 3 | RS | 53 | ?recurr | – | Laparotomy |
| 4 | RN | 66 | Ca | Hep flex | Rt Hemi |
| 5 | DN | 51 | Ca | Hep flex | Rt Hemi |
| 6 | JL | 71 | Ca | Rectum | Ant resect. |
| 7 | JH | 79 | Ca | Trans | Lt Hemi |
| 8 | BT | 58 | Ca | Rectum | Ant resect. |
| 9 | JP | 59 | Ca | Sigmoid | Lt Hemi |
| 10 | KH | 65 | ?recurr | – | – |
| 11 | MJ | 67 | Ca | Sigmoid | Lt Hemi |
| 12 | BB | 54 | Ca | Rectum | Ant resect. |
| 13 | VD | 74 | Ca | Sigmoid | Lt Hemi |
| 14 | FH | 76 | Ca | Sigmoid | Lt Hemi |
| 15 | BS | 60 | Ca | Sigmoid | Lt Hemi |
| 16 | RD | 64 | Lipoma | Sigmoid | – |
| 17 | JP | 76 | Ca | Spl flex | Lt Hemi |
| 18 | EB | 73 | Adenoma | Caecum | Rt Hemi |
| 19 | DN | 73 | Ca | Sigmoid | Lt Hemi |
| 20 | DF | 79 | Adenoma | Rectum | Laser |
| 21 | GX | 67 | Ca | R/S | Ant resect. |
| 22 | JB | 56 | Ca | Sigmoid | Ant resect. |

No.: number in series; Init.: patients initials; Ca: carcinoma; ?recurr: suspected recurrent tumour; R/S: rectosigmoid; Hep flex: hepatic flexure; Trans: transverse colon; Spl flex: splenic flexure; Lt Hemi: left hemicolectomy; Rt Hemi: right hemicolectomy; Ant resect: anterior resection.

### *Imaging and organ uptake*

Images were obtained of the anterior and posterior abdomen and pelvic outlet at 10 min and 24, 48 and 72 h after administration of $^{111}$In-ICR2 using a gamma camera (Siemens) fitted with a medium energy collimator and connected to a computer for data analysis and information storage (Nodecrest). A positive tumour localisation was assessed as a focal uptake seen in the images at 24, 48 and 72 h which was not present on the blood pool image and which corresponded to a known tumour site on subsequent laparotomy.

The gamma camera was also used to assess the uptake of radiolabelled antibody in the liver, spleen, kidneys and bone marrow using an anthropomorphic phantom and region of interest (ROI) analysis for the liver and ROI analysis alone for the spleen, kidneys and bone marrow (Davidson, 1989*b*).

### *Determination of EMA in serum*

Normal sera contains EMA or EMA like substances that bind to ICR2. To determine the amount in the patients sera a competitive radioimmunoassay was used that is based on the binding of $^{125}$I-ICR2 to plates coated with purified EMA (Ormerod *et al.*, 1983). Serial dilutions of sera in phosphate buffered saline containing 0.5% bovine serum albumin (PBS/BSA) were made in triplicate and 30 μl aliquots of each were mixed with an equal volume of $^{125}$I-ICR2 (105 c.p.m. $MBq^{-1}$). Aliquots of 50 μl of the mixture were then transferred to 96 well plates (Nunc) that had been pretreated with EMA by covering each well with 50 μl of PBS/BSA containing 5 $\mu g\ ml^{-1}$ of EMA and then blocking for 1 h at 37°C with 200 μl well of PBS/BSA. Unbound radioactivity was removed by repeated washing with PBS-BSA and then the bound $^{125}$I-ICR2 detected by counting each well in an Innotron Hydragamma 16 well counter (John Cant, Evesham, UK). Standard curves were prepared by mixing known concentrations of EMA with $^{125}$I-ICR2 and incubating with the EMA coated multiwell plates.

### *Circulating levels of immune complex*

The levels of immune complex in the serum were measured at intervals after the administration of radiolabelled antibody by high performance liquid chromatography (HPLC) on a Zorbax GF250 (DuPont, Massachussetts). The serum taken from patients before administration of the radiolabelled antibody was also tested by HPLC for immune complex formation with $^{125}$I-ICR2. $^{125}$I-ICR2 was added to the serum to give a concentration of 0.3 $\mu g\ ml^{-1}$ which is equivalent to the concentration found by administering 1 mg of MAb to a patient with a plasma volume of 3 litres (blood volume of 5 litres and haematocrit of 40%).

### *Blood clearance, urinary excretion and tumour uptake*

Following administration the clearance of radiolabelled antibody from the blood was determined by gamma well counting of paired 2 ml serum samples taken at 10 min, 30 min, 24, 48 and 72 h.

The urinary excretion and uptake of radioactivity by the tumour was calculated as a percentage of the injected dose of radioactivity (% I.D.) by preparing a known dilution of the injectate and determining its radioactivity at the same time as that of the sample of tumour or urine being measured, again using a gamma well counter.

### Results

### In vitro *analysis*

When $^{125}I$ or $^{111}In$-labelled ICR2 which has been prepared for *in vitro* or clinical studies was analysed by HPLC it was found to elute as a single peak corresponding to a molecular weight of 150–160,000. When serum samples from either healthy volunteers or patients with cancer were mixed with either of the radiolabelled antibodies two peaks eluted from the column. The first, comprising approximately 30% of the labelled activity, eluted within the void volume of the column and had a molecular weight > 600,000. The second peak contained the remainder and corresponded to the molecular weight expected for IgG (150–160,000).

With all serum samples the amount of high molecular weight complex formed with radiolabelled antibody was reduced by first incubating the serum with unlabelled antibody. The percentage of $^{125}I$ or $^{111}In$ labelled ICR2 present in the complex was reduced with progressive increase in the concentration of the cold antibody. In the example shown in Figure 1(a–d) immune complex was reduced from 37.6% of the total activity at a ratio of unlabelled to labelled of 1:1 (1a) to 5.7% at a 100:1 ratio (1d).

**Figure 1(a–d)** The effect of cold antibody administration on immune complex formation. HPLC analysis of human serum following reaction with $^{111}In$-ICR2 demonstrated a high molecular weight peak formed by immune complex and a low molecular weight peak formed by monomeric IgG. The effect of adding cold unlabelled antibody to the serum prior to radiolabelled MAb administration at concentrations of 1:1 **a**, 3:1 **b**, 10:1 **c**, and 100:1 **d** are shown. The area under the curve has been integrated to give the percentage of the total circulating activity forming the high molecular weight complex.

### Clinical studies

### *Imaging results*

Of the 22 patients to whom radiolabelled MAb was administered two were not imaged, three had benign tumours and two were shown not to have recurrent colorectal cancers. Of the 15 patients with primary cancers the number of tumour sites localised were not significantly different in control group (Group A) (five or six + ve), the group given antibody pretreated with chelate (Group B) (four of six + ve) or the group pretreated with unlabelled antibody (Group C) (three of three + ve). Similarly the level of background radioactivity in the images produced was not found to be different in an objective assessment by a nuclear medicine clinician.

### *Blood clearance*

The clearance of radioactivity from the blood is shown as a percentage of the level at 10 min in Figure 2. Here, the mean effective half life of the circulating activity was 39 h for controls (Group A), 33 h for the Group B given chelate treated antibody and 27 h for Group C pretreated with cold antibody. Furthermore the level of radioactivity in the blood was significantly less in Group B at 48 h ($P = 0.005$) and in Group C at both 24 h ($P = 0.034$) and 48 h ($P = 0.000$) compared with controls. We conclude that the rate of clearance of antibody from the blood was enhanced by pretreatment of radiolabelled antibody with DTPA or by pretreatment of the patient with unlabelled ICR2.

**Figure 2** Blood clearance of radioactivity. The level of radioactivity in the circulation with time is shown compared with samples taken at 10 min after radiolabelled MAb administration. The results are mean ± standard deviation for the controls, chelate and cold antibody groups.

*Urinary excretion*

The urinary excretion of radioactivity was significantly reduced over the first 12 h period when DTPA treated $^{111}$In-ICR2 was administered (16.2 ± 6.94 *vs* 2.9 ± 1.8% of injected dose, $P = 0.007$) but excretion was not affected by the administration of unlabelled ICR2.

*Tumour uptake*

The absolute uptake of activity in the tumours as determined by gamma well counting of specimens obtained at surgery ranged from 0.0016% I.D. $g^{-1}$ to 0.016% I.D. $g^{-1}$ with a tumour to normal colon ratio of 2.1 ± 0.915 (mean ± s.d.). There was no significant difference in the value between the three groups of patients.

*Organ uptake of radiolabelled antibody*

By 48 h after administration about 20% of the injected dose had accumulated in the liver of patients in the control group A (21.9 ± 4.6, Table IIa). An increased percentage of the injected dose was found to accumulate in the liver of patients in group B given antibody pretreated with DTPA at 45 min (22.2 ± 2.6 *vs* 18.1 ± 3.2, $P = 0.049$), 24 (26.0 ± 4.9 *vs* 20.7 ± 3.5, $P = 0.029$) and 48 h (31.6 ± 3.7 *vs* 21.9 ± 4.6, $P = 0.002$). The preadministration of cold MAb to patients in Group C led to a reduced liver uptake at 48 h (26% *vs* 32%, $P < 0.05$) but no significant differences were observed at the earlier times.

By 48 h after administration 2–3% of the injected dose had accumulated in the spleen in the control group (2.44 ± 1.4). Analysis of the counts accumulating in the spleen per MBq of injected activity showed this to be significantly increased in the other groups (Group B, $P = 0.05$) (Group C, $P < 0.05$) (Table IIb). No significant differences were found between the groups regarding the renal uptake or that of the bone marrow (data not shown).

*Analysis of immune complexes* in vivo

Serum from 16 of the 22 patients undergoing immunoscintigraphy were analysed by HPLC. When these sera were reacted *in vitro* with $^{125}$I-ICR2, a mean of 31.5 ± 3.6% of the radioactivity was found in the immune complex of > 600 kD. When serum samples taken from the patients were analysed by HPLC the amount of the high molecular weight circulating immune complex was found to decrease with time after administration of the radiolabelled ICR2. When 5 mg of cold MAb was injected before the administration of the radiolabelled antibody only a small reduction in the quantity of immune complex was observed from 34.2% to 30.2% of the circulating radioactivity.

*Circulating antigen levels*

The apparent levels of EMA in the patients serum varied from 60–1150 ng $ml^{-1}$ with a mean level in the total patient group of 544 ± 340 ng $ml^{-1}$. No significant differences were noted between the rate of clearance of $^{111}$In-ICR2 from the blood of patients with low ($n = 9$, < 500 ng $ml^{-1}$) or high ($n = 6$, > 500 ng $ml^{-1}$) levels of circulating antigen. A strong correlation was found, however, between the level of EMA in the patients serum prior to $^{111}$In-ICR2 administration and the amount of immune complex formed following administration, patients with higher circulating antigen levels forming greater amounts of circulating immune complex ($n = 11$), $r = 0.61$, $P < 0.05$) (Figure 3). Circulating antigen levels also had a direct correlation with tumour uptake of radiolabelled antibody ($n = 9$, $r = 0.65$, $P = 0.05$) (Figure 4).

**Discussion**

This study has shown that when the anti-EMA antibody ICR2 was injected intravenously into patients with colon cancer a high molecular weight immune complex was formed in the blood with antigen or antigen-like material.

Studies *in vitro* show that the same high molecular weight complex was formed when the anti-EMA antibody ICR2, labelled with either $^{111}$In or $^{125}$I, was mixed with serum from patients with colon cancer or normal controls. The reduction in amount of radiactivity present in the high molecular weight complex by the addition of cold antibody would suggest that it is a true immune complex.

It would seem likely, although it is rarely reported, that radiolabelled monoclonal antibodies raised against other secreted tumour associated antigens (NIH consensus statement 1981; Magnani *et al.*, 1983; Canney *et al.*, 1984; Nouwen *et al.*, 1985; Chia *et al.*, 1985; Hilkens *et al.*, 1986; Klug *et al.*, 1986; Ashorn *et al.*, 1988; Ben-Mahrez *et al.*, 1988) would also form circulating immune complexes when administered for immunoscintigraphy (Mach *et al.*, 1980*a*; Primus *et al.*, 1980; Dillman *et al.*, 1984; Zalutsky *et al.*, 1988). Although it has been suggested by some authors that the presence of antigen in the blood does not significantly affect the results of imaging (Goldenberg *et al.*, 1978; Mach *et al.*, 1980*b*) other studies have shown that when an increased dose of antibody has been administered the number

**Table II** Organ uptake of radioactivity

(a) Liver

| | Patient group | | |
|---|---|---|---|
| *Time after administration* | *Control (A)* | *Chelate (B)* | *Cold antibody (C)* |
| 45 min | 18.1±3.2 | 22.2±2.6[a] | 18.7±6.0 |
| 24 h | 20.7±3.5 | 26.0±4.9[b] | 22.8±4.4 |
| 48 h | 21.9±4.6 | 31.6±3.7[c] | 25.8±4.0[d] |

Organ uptake as a percentage of the injected dose (mean ± standard deviation). Statistical comparison with controls [a]$P = 0.049$; [b]$P = 0.029$; [c]$P = 0.002$, [d]Cold MAb *vs* chelate alone $P < 0.05$.

(b) Spleen

| | Patient group | | |
|---|---|---|---|
| *Time after administration* | *Control (A)* | *Chelate (B)* | *Cold antibody (C)* |
| 45 min | 433±154 | 603±124[a] | 815±157[b] |
| 24 h | 470±159 | 615±141[a] | 859±110[b] |
| 48 h | 450±149 | 526±76[a] | 800±143[b] |

Uptake as counts/5 min/MBq of injected activity (mean ± standard deviation). Statistical comparison with controls. [a]$P = 0.05$; [b]$P < 0.05$.

**Figure 3** Circulating antigen and immune complex formation. Circulating antigen levels were measured by radioimmunoassay in serum samples taken prior to radiolabelled MAb administration and the amount of immune complex formed was calculated by HPLC of serum following radiolabelled antibody administration. The relationship is shown between circulating antigen levels and immune complex formation.

**Figure 4** Circulating antigen levels and the tumour uptake of radiolabelled MAb. The circulating antigen levels were measured by radioimmunoassay. Tumour uptake of radiolabelled MAb was calculated by weighing and gamma well counting biopsies of resected tumour and comparing the radioactivity per gram to a standard produced as a known percentage of the injected radioactivity. The association between circulating antigen in $ng\,ml^{-1}$ and the tumour uptake as a percentage of the injected dose is shown.

of tumour sites detected on immunoscintigraphy is increased (Halpern *et al.*, 1985; Murray *et al.*, 1985; Carrasquillo *et al.*, 1986). This conflict of opinion on the significant of circulating immune complexes may be explained by differences in the antigen associated with the tumour and those present in the circulation and also in their affinity of binding to administered radiolabelled antibody. Such differences explain variations in the reported value of administering unlabelled MAb during immunoscintigraphy.

Based on the *in vitro* data a group of patients in the present study were given a small dose (5 mg) of unlabelled ICR2 prior to injection of the $^{111}$In labelled antibody. This treatment did not, however, result in a significant improvement in the number of tumour deposits localised on imaging, nor did it enhance the tumour to background ratio of radioactivity or lead to a greater uptake of radioactivity by the tumours. This finding contrasts with those of previous studies where it was concluded that administration of cold antibody was beneficial. The earlier studies, however, claimed an improvement only in the number of tumour deposits localised in patients with multiple tumours and have not demonstrated increased tumour uptake of radioactivity (Halpern *et al.*, 1985; Murray *et al.*, 1985; Carrasquillo *et al.*, 1986).

The biodistribution analysis suggests a reason why the tumour uptake of radiolabelled MAb was not significantly increased by the preadministration of unlabelled antibody in that it was found that the percentage of radioactivity forming immune complexes in the blood was reduced by only 4% following administration of 5 mg of unlabelled antibody. A large dose of cold antibody would therefore be required to prevent or significantly reduced the formation of immune complexes. A better understanding of the importance of circulating antigen in immunoscintigraphy would be achieved if accurate analysis of the biodistribution of radiolabelled antibody was performed routinely.

Clearly the problems associated with circulating antigen need to be overcome. One approach would be to use antibodies to tumour associated antigens which are not secreted or alternatively antibodies which recognise epitopes not present on the secreted antigen molecule.

A correlation was found between the level of circulating antigen and the uptake of radiolabelled ICR2 into the immune complexes, those patients with highest levels of circulating antigen having the greatest amount of circulating immune complex in the blood. However, the strong correlation between the amount of circulating immune complex present in the blood and the uptake of radioactivity by the tumour would not have been expected and has not been reported previously. This finding would suggest that patients with large amounts of circulating immune complexes and small amounts of free radiolabelled antibody have the best uptake of radiolabelled antibody into their tumours. The relationship between the expression of antigen at the tumour cell surface ad the amount shed into the circulation is controversial and is likely to vary from one tumour associated antigen to another (Wagener *et al.*, 1981; Martin & Halpern, 1984; Philben *et al.*, 1986). It is possible that satisfactory antibody imaging may be achieved despite high levels of circulating antigen if the tumour associated antigen is strongly expressed on the cell surface or the affinity of the antibody was greater for the tumour bound antigen than to that in the circulation.

The pretreatment of the radiolabelled antibody with chelate has not been found to be of any value in this clinical study. The uptake of radiolabelled antibody in the liver and spleen was greater in the group receiving chelate treated antibody than in the untreated controls, suggesting that this uptake of activity is largely due to stable radiolabelled antibody rather than dissociated $^{111}$In. This result contrasts with the situation *in vitro* where a significant reduction in the uptake of $^{111}$In-MAb by hepatocytes was achieved using chelating agents without reducing the tumour uptake (Davidson *et al.*, 1990). The difference between the results found *in vitro* and in the clinical investigation may, however, be due to differences in the concentration of chelate in the environment of the hepatocytes, a high local concentration of chelate being required to prevent the uptake of $^{111}$In by hepatocytes. The concentration required to prevent hepatocyte uptake *in vitro* would not be safely obtainable *in vivo*. Indeed, studies on the use of chelating agents to inhibit the liver uptake of radiolabelled antibodies have been carried out in animals but the results have failed to establish a useful role for this procedure (Ward *et al.*, 1986).

Before immunoscintigraphy can become routinely employed there are a number of problems which must be addressed. Firstly there is a need for antibodies with a greater tumour specificity so that the uptake by normal tissues is reduced. Second, it is essential to lower the non specific uptake and retention of radionuclide by organs such as the liver. Here the use of Iodine-124 or $^{99m}$Tc would be expected to lead to substantial improvements (Goldenberg *et al.*, 1990; Lamki *et al.*, 1990). The use of bispecific antibodies which recognise both a tumour antigen and a low molecular weight probe that may be radiolabelled also offers a number of advantages (Goodwin *et al.*, 1988), not least that administration of the radiolabelled probe can be done after antibodies that are non specifically bound to normal tissue are cleaved from the cell surface. Other techniques have been employed to improve immunoscintigraphy, Paik and colleagues (1989) describing the use of a metabolisable linker joining the MAb with the radiolabel and showing increased tumour to background ratios in an animal xenograft model. The use of the biological linkage of avidin and biotin has also been investigated although the results of these studies remain preliminary (Kalofonos *et al.*, 1990). Other methods for optimising immunoscintigraphy are required along with careful evaluation of the results.

### References

ARMITAGE, N.C., PERKINS, A.C., PIMM, M.V. & 5 others (1986). Imaging of bone tumors using a monoclonal antibody raised against human osterosarcoma. *Cancer*, **58**, 37.

ASHORN, P., KALLIONIEMI, O.-P., HIETANEN, T., ASHORT, R. & KROHN, K. (1988). Elevated serum HMFG antigen levels in breast and ovarian cancer patients measured with a sandwich elisa. *Int. J. Cancer*, (Suppl 2), 28.

BEN-MAHREZ, K., THIERRY, D., SOROKINE, I., DANNA-MULLER, A. & KOHIYAMA, M. (1988). Detection of circulating antibodies against c-myc protein in cancer patient sera. *Br. J. Cancer*, **57**, 529.

CANNEY, P.A., MOOR, M., WILKINSON, P.M. & JAMES, R.D. (1984). Ovarian cancer antigen CA125: a prospective clinical assessment of its role as a tumour marker. *Br. J. Cancer*, **50**, 765.

CARRASQUILLO, J.A., BUNN, P.A., KEENAN, A.M. & 12 others (1986). Radioimmunodetection of cutaneous T-cell lymphoma with In111 labelled T101 monoclonal antibody. *N. Engl. J. Med.*, **315**, 673.

CARRASQUILLO, J.A., MULSHINE, J.L., BUNN, P.A. & 8 others (1987). Indium-111 T101 monoclonal antibody is superior to Iodine-131 T101 in imaging of cutaneous T-cell lymphoma. *J. Nucl. Med.*, **28**, 281.

CARRASQUILLO, J.A., ABRAMS, P.G., SCHROFF, R.W. & 12 others (1988). Effect of antibody dose on the imaging and biodistribution of indium-111 9.2.27 anti-melanoma monoclonal antibody. *J. Nucl. Med.*, **29**, 39.

CHAN, S., EVAN, G., RITSON, A., WATSON, J., WRAIGHT, P. & SIKORA, K. (1986). Localisation of lung cancer by a radiolabelled MCA against the C-myc oncogene product. *Br. J. Cancer*, **54**, 761.

CHATAL, J.-F., SACCAVINI, C.R., FUMOLEAU, P. & 4 others (1984). Immunoscintigraphy of colon carcinoma. *J. Nucl. Med.*, **25**, 307.

CHIA, D., TERASAKI, P.I., SUYAMA, N., GALTON, J., HIROTA, M. & KATZ, D. (1985). Use of monoclonal antibodies to sialylated Lewis$^{x}$ and sialylated Lewis$^{a}$ for serological tests for cancer. *Cancer Res.*, **45**, 435.

DAVIDSON, B.R., YIU, C.Y., STYLES, J., ORMEROD, M., CLARK, C.G. & DEAN, C. (1988). A comparison of carcinoembryonic antigen (CEA) and epithelial membrane antigen (EMA) in human colorectal cancer. *Int. J. Cancer*, (Suppl 3), 56.

DAVIDSON, B.R., SAMS, V., STYLES, J., DEAN, C. & BOULOS, P.B. (1989*a*). Comparative study of carcinoembryonic antigen and epithelial membrane antigen expression in normal colon, adenomas and adenocarcinomas of the colon and rectum. *Gut*, **30**, 1260.

DAVIDSON, B.R. (1989*b*). The role of monoclonal antibodies to epithelial membrane antigen (EMA) in the investigation of patients with colorectal cancer. *M D Thesis*, University of Glasgow.

DAVIDSON, B.R., BOULOS, P.B. & PORTER, J. (1990). Radiolabelled antibody imaging of gastrointestinal cancers: can chelating agents improve the results obtained? *Eur. J. Nucl. Med.*, **17**, 294.

DILLMAN, R.O., BEAUREGARD, J.C., SOBUL, R.E. & 4 others (1984). Lack of radioimmunodetection and complications associated with monoclonal anticarcinoembryonic antigen antibody cross reactivity with an antigen on circulating cells. *Cancer Res.*, **44**, 2213.

GOLDENBERG, D.M., DELAND, F., KIM, E. & 6 others (1978). Use of radiolabelled antibodies to carcinoembryonic antigen for the detection and localisation of diverse cancers by external photoscanning. *New Engl. J. Med*, **298**, 1384.

GOLDENBERG, D.M., GOLDENBERG, H., SHARKEY, R.M. & 9 others (1990). Clinical studies of cancer radioimmunodetection with carcinoembryonic antigen monoclonal antibody fragments labelled with 123I or 99mTc. *Cancer Res.*, **50** (Suppl 3), 909s.

GOODWIN, D.A., MEARES, C.F., MCTIGUE, M., MCCALL, M.J. & CHAOVAPONG, G.W. (1986). Metal decomposition rates of $In^{111}$ DTPA and EDTA conjugates of monoclonal antibodies *in vivo*. *Nucl. Med. Communic.*, **7**, 831.

GOODWIN, D.A., MEARES, C.F., MCCALL, M.J., MCTIGUE, M. & CHAOVAPONG, W. (1988). Pre-targeted immunoscintigraphy of murine tumors with Indium-111-labeled bifunctional haptens. *J. Nucl. Med.*, **29**, 226.

HALPERN, S.E., HAGAN, P.L., GARVER, P.R. & 6 others (1983). Stability, characteristics and kinetics of 111In labeled monoclonal antitumor antibodies in normal animals and nude mouse-human tumor models. *Cancer Res.*, **43**, 5347.

HALPERN, S.E., DILLMAN, R.O., WITZTUM, K.F. & 10 others (1985). Radioimmunodetection of melanoma utilising In-111 96.5 monoclonal antibody: a preliminary report. *Radiology*, **155**, 493.

HILKENS, J., KROEZEN, V., BONFRER, M., BAKKER, DE-J. & BRUNING, P.F. (1986). MAM-6 Antigen, a new serum marker for breast cancer monitoring. *Cancer Res.*, **46**, 2582.

HNATOWICH, D.J., GRIFFIN, T.W., KOSCIUCZYK, C. & 5 others (1985). Pharmacokinetics of an Indium 111 labelled monoclonal antibody in cancer patients. *J. Nucl. Med.*, **26**, 849.

HNATOWICH, D.J. & MCGANN, J. (1987). DTPA-coupled proteins-procedures and precautions. *Nucl. Med. Biol.*, **14**, 563.

IMRIE, S.F., SLOANE, J.P., ORMEROD, M.G., STYLES, J. & DEAN, C.J. (1990). Detailed investigation of the diagnostic value in tumour histopathology of ICR.2, a new monoclonal antibody to epithelial membrane antigen. *Histopathology*, **16**, 573.

KALOFONOS, K.P., RUSCKOWSKI, M., SIEBECKER, D.A. & 5 others (1990). Imaging of tumor in patients with Indium-111-labeled Biotin and Streptavidin-conjugated antibodies: preliminary communication. *J. Nucl. Med.*, **31**, 1791.

KLUG, T.L., SATTLER, M.A., COLCHER, D. & SCHLOM, J. (1986). Monoclonal antibody immunoradiometric assay for an antigenic determinant (CA 72) on a novel pancarcinoma antigen (TAG 72). *Int. J. Cancer*, **38**, 661.

KREJCAREK, G.E. & TUCKER, K.L. (1977). Covalent attachment of chelating groups to macromolecules. *Biochem. Biophys. Res. Comm.*, **77**, 581.

LAMKI, L.M., MURRAY, J.L., ROSENBLUM, M.G., PATT, Y.Z., BABAIAN, R. & UNGER, M.W. (1988). Effect of unlabelled monoclonal antibody (MoAb) on biodistribution of 111-Indium labelled MoAb. *Nucl. Med. Communic.*, **9**, 553.

LAMKI, L.M., ZUKIWSKI, A.A., SHANKEN, L.J. & 6 others (1990). Radioimaging of melanoma using 99m Tc-labeled FAB fragment reactive with a high molecular weight melanoma antigen. *Cancer Res.*, **50** (Suppl 3), 904s.

MACH, J.P., FORNI, M., RITSCHARD, J., BUCHEGGER, F., CARREL, S., WIDGREN, S., DONATH, A. & ALBERTO, P. (1980*a*). Use and limitations of radiolabelled anti-CEA antibodies and their fragments for photoscanning detection of human colorectal carcinomas. *Oncodev. Biol. Med.*, **1**, 49.

MACH, J.P., CARREL, S., FORNI, M., RITSCHARD, J., DONATH, A. & ALBERTO, P. (1980*b*). Tumour localisation of radiolabelled antibodies against carcinoembryonic antigen in patients with carcinoma. *N. Engl. J. Med.*, **303**, 5.

MAGNANI, J.L., STEPLEWSKI, Z., KOPROWSKI, H. & GINSBURG, V. (1983). Identification of the gastrointestinal and pancreatic cancer associated antigen detected by monoclonal antibody 19–9 in the sera of patients as a mucin. *Cancer Res.*, **43**, 5489.

MARTIN, K.W. & HALPERN, S.E. (1984). Carcinoembryonic antigen production, secretion and kinetics in BALB/c mice and a nude mouse-human tumor model. *Cancer Res.*, **44**, 5475.

MURRAY, J.L., ROSENBLUM, M.G., SOBOL, R.E. & 13 others (1985). Radioimmunoimaging in malignant melanoma with 111In labelled monoclonal antibody 96.5. *Cancer Res.*, **45**, 2376.

NOUWEN, E.J., POLLET, D.E., SCHELSTRAETE, J.B. & 4 others (1985). Human placental alkaline phosphatase in benign and malignant ovarian neoplasia. *Cancer Res.*, **45**, 892.

NIH CONSENSUS STATEMENT (1981). Carcinoembryonic antigen: its role as a marker in the management of cancer. *Br. Med. J.*, **282**, 373.

ORMEROD, M.G., STEELE, K., WESTWOOD, J. & MAZZINI, M.N. (1983). Epithelial membrane antigen: partial purification, assay and properties. *Br. J. Cancer*, **48**, 533.

PAIK, C.H., YOKOYAMA, K., REYNOLDS, J.C & 6 others (1989). Reduction of background activities by introduction of a diester linkage between antibody and a chelate in radioimmunodetection of tumor. *J. Nucl. Med.*, **30**, 1693.

PHILBEN, V.J., JAKOWATZ, J.G., BEATTY, B.G. & 5 others (1986). The effect of tumor CEA content and tumor size on tissue uptake of Indium 111 labeled anti-CEA monoclonal antibody. *Cancer*, **57**, 571.

PRIMUS, F.J., BENNETT, S.J., KIM, E., DELAND, F.H., ZAHN, M.C. & GOLDENBERG, D.M. (1980). Circulating immune complexes in cancer patients receiving goat radiolocalising antibodies to carcinoembryonic antigen. *Cancer Res.*, **40**, 497.

RAINSBURY, R.M., OTT, R.J., WESTWOOD, J.H. & 5 others (1983). Location of metastatic breast carcinoma by a monoclonal antibody chelate labelled with Indium-111. *Lancet*, **ii**, 934.

RAINSBURY, R.M. (1984). The localisation of human breast carcinomas by radiolabelled monoclonal antibodies. *Br. J. Surg.*, **71**, 805.

ROSENBLUM, M.G., MURRAY, J.L., HAYNIE, T.P. & 6 others (1985). Pharmacokinetics of 111In labelled anti-p97 monoclonal antibody in patients with metastatic malignant melanoma. *Cancer Res.*, **45**, 2383.

SHEPHERD, J.H., GRANOWSKA, M., BRITTON, K.E. & 4 others (1987). Tumour-associated monoclonal antibodies for the diagnosis and assessment of ovarian cancer. *Br. J. Obstet. Gynaecol.*, **94**, 160.

WAGENER, C., MULLAR-WALLRAF, R., NISSON, S., GRONER, J. & BREUER, H. (1981). Localization and concentration of carcinoembryonic antigen (CEA) in gastrointestinal tumors: correlation with CEA levels in plasma. *J.N.C.I.*, **67**, 539.

WARD, M.C., ROBERTS, K.R., WESTWOOD, J.H., COOMBES, R.C. & MCCREADY, V.R. (1986). The effect of chelating agent on the distribution of monoclonal antibodies in mice. *J. Nucl. Med.*, **27**, 1746.

ZALUTSKY, M.R., KNAPP, R.C. & BAST, R.C. (1988). Influence of circulating antigen on blood pool activity of a radioiodinated monoclonal antibody. *Nucl. Med. Biol.*, **15**, 431.